# Predicting the Maximum Earthquake Magnitude from Seismic Data in Israel and Its Neighboring Countries

**DOI:** 10.1371/journal.pone.0146101

**Published:** 2016-01-26

**Authors:** Mark Last, Nitzan Rabinowitz, Gideon Leonard

**Affiliations:** 1 Department of Information Systems Engineering, Ben-Gurion University of the Negev, Beer-Sheva, Israel; 2 Human Monitoring Ltd., Rehovot, Israel; 3 Israel Atomic Energy Commission, Tel-Aviv, Israel; Qom University, ISLAMIC REPUBLIC OF IRAN

## Abstract

This paper explores several data mining and time series analysis methods for predicting the magnitude of the largest seismic event in the next year based on the previously recorded seismic events in the same region. The methods are evaluated on a catalog of 9,042 earthquake events, which took place between 01/01/1983 and 31/12/2010 in the area of Israel and its neighboring countries. The data was obtained from the Geophysical Institute of Israel. Each earthquake record in the catalog is associated with one of 33 seismic regions. The data was cleaned by removing foreshocks and aftershocks. In our study, we have focused on ten most active regions, which account for more than 80% of the total number of earthquakes in the area. The goal is to predict whether the maximum earthquake magnitude in the following year will exceed the median of maximum yearly magnitudes in the same region. Since the analyzed catalog includes only 28 years of complete data, the last five annual records of each region (referring to the years 2006–2010) are kept for testing while using the previous annual records for training. The predictive features are based on the Gutenberg-Richter Ratio as well as on some new seismic indicators based on the moving averages of the number of earthquakes in each area. The new predictive features prove to be much more useful than the indicators traditionally used in the earthquake prediction literature. The most accurate result (AUC = 0.698) is reached by the Multi-Objective Info-Fuzzy Network (M-IFN) algorithm, which takes into account the association between two target variables: the number of earthquakes and the maximum earthquake magnitude during the same year.

## Introduction

As indicated by [1], the concept of time-dependent seismicity, which implies that current seismicity should be evaluated on the basis of the past data, has become an important topic in the evaluation of seismic hazards. Generally, there are two different aspects of earthquake prediction: *long-term forecasting* and *short-term forecasting* [2]. Whereas short-term forecasting is supposed to predict the exact time, location, and magnitude of an earthquake event, we focus here on long-term forecasting, which aims at predicting a large earthquake a year or even several years in advance. According to [2], most existing methods of long-term earthquake prediction are looking for *recurrence patterns* in the sequence of tectonic events occurring in the same region. However, large earthquakes often fail to occur around their expected recurrence times [3].

It has long been observed that the relationship between the frequency and the magnitude of seismic events in a given region follows the power law [4]. In other words, when observing earthquake occurrences over time we expect the earthquakes of small magnitude to be much more frequent than the earthquakes of large magnitude. Following the study of California earthquakes by [5], this relationship is named the Gutenberg-Richter inverse power law and it is defined mathematically as follows:

$${\text{log}}_{10}N(M)=a-bM$$ (1)

where *N(M)* is the cumulative number of events with magnitude greater or equal to *M*, *a* represents the logarithm of the number of earthquakes with magnitude greater or equal to zero, and *b* is the slope of the above equation. The values of *a* and *b* can be estimated from an empirical distribution of earthquake frequencies using the linear regression analysis [4]. The power law model is widely used for building earthquake hazard maps [6], which estimate the probability of exceeding a given magnitude over a specified amount of time, usually tens or hundreds of years. Thus, it is usually applied to long-term earthquake forecasting (like in [7]).

A long-term forecasting model for estimating large (M ≥ 4.95) earthquake probabilities is presented in [8]. Their model extends the common recurrence approach by assuming that future earthquakes are more likely to occur in areas where past earthquakes have occurred, including small ones (M ≥ 2). This assumption implies that the future number of earthquakes in a given area can be predicted based on the past number of earthquakes in the neighboring locations. They used the records of 300,278 seismic events in California from 1 January 1981 until 1 April 2010 with magnitude M ≥ 1.7. Their predictive feature is the *seismicity rate* (the number of M ≥ 2 events in a given cell smoothed by a kernel function) during the 24-year training period, whereas the predicted attribute is the number of M ≥ 3.5 or M ≥ 4.95 events during the subsequent five-year target period. In their experiments, the number of neighboring locations (the smoothing parameter) was set to 10. The effect of more distant locations was completely ignored. The magnitude distribution in a given location (cell) during the target period was estimated using the Gutenberg—Richter (GR) ratio [5]. The performance of each prediction model was evaluated by its average probability gain per earthquake relative to the reference (uniform) model. The highest probability gain reported by [8] was about 6.0.

Another attempt to utilize recurrence patterns in seismic time series is made in [9]. The goal is to predict events with a magnitude greater or equal to 4.5. The data points in a given area are grouped into sets of five chronologically ordered earthquakes. Each sequence is represented by the mean of the magnitude of the five-earthquakes, the time elapsed from the first earthquake and the fifth one and the signed variation of the *b*-values in the Gutenberg—Richter equation, which is determined from the fifty preceding events. Based on these three features, the five-event sequences in each area are partitioned into groups using the *k*-means clustering algorithm [10]. The authors have used the Spanish seismic data set, which included 4,017 earthquakes, whose magnitude varied between 3.0 and 7.0, during a 29-year period (1978–2007). The optimal number of clusters (*k*) was set to three according to the silhouette index [11]. After applying the proposed methodology to two separate areas in the dataset, having the highest amount of seismic events, certain sequences of clusters were found as precursors of moderate—large earthquakes with sensitivity and specificity of about 80%-90%.

The study of [4] makes an attempt to utilize multiple predictive features for predicting the magnitude of the largest seismic event in the following month, which is considered a short-term forecasting task. Their feature set contains eight seismic indicators including three indicators based on the Gutenberg—Richter law. Three neural network models were used in their study: a feed-forward Levenberg-Marquardt backpropagation (LMBP) neural network, a recurrent neural network, and a radial basis function (RBF) neural network. The models were trained and tested on seismic data from two different regions in California. In each region, the training set included monthly data from 41 years (1950–1990), whereas the period from January 1991 to September 2005 was used for testing. The monthly seismicity indicators were calculated for sliding windows of the last 50, 100, and 200 seismic events. Six binary classification tasks were defined by increasing the threshold magnitude from 4.5 to 7.0 in increments of 0.5. The network output of 1 represented the occurrence of an earthquake of predefined threshold magnitude or greater during the following month. The output of 0 stood for an absence of such an event. For the lowest threshold of 4.5, the reported recall (“hit rate”) of earthquakes exceeding the threshold magnitude varied between 0.44 and 0.67 with the false alarm rate of 0.31–0.44.

Though the level of seismic activity in Israel and its neighboring countries is considered moderate, the region did experience some devastating earthquakes in the past and the rapid increase in population density, along with inconsistent construction standards, makes it particularly vulnerable to strong earthquakes in the future [7]. The authors of [1] have studied the earthquake distribution along the Dead Sea Fault (DSF) during the past 60,000 years. They analyzed data sets of prehistoric—paleoseismic, historical, and modern (instrumental) observations (from the years 1983–2007) in three different segments along the DSF: the southern Arava Valley, the northern Jordan Valley, and the Dead Sea basin. They found that the Gutenberg—Richter relation between the magnitude and frequency of earthquakes [5] provides a good explanation for the data observed in each segment separately as well as for all DSF data. The Gutenberg—Richter relation was also used by [7] for estimating the earthquake recurrence rates and the 50-year seismic hazard for ten seismic zones in Israel.

This paper presents the first attempt to apply data mining and time-series analysis methods for long-term earthquake prediction in Israel and its vicinity, utilizing a relatively small catalog of seismic events. We also construct a larger set of seismic indicators than the previous works in long-term earthquake forecasting and perform a comprehensive evaluation of multiple classification techniques on this task. Rather than training separate classification models on small datasets related to each region, we combine the data of 10 regions into one data table to produce a general classification model. Then we demonstrate the use of ROC curves for evaluating earthquake forecasting methods. We show that the Multi-Objective Info-Fuzzy Network (M-IFN) algorithm outperforms single-objective classification algorithms on the earthquake prediction task. Finally, we indicate further directions for the contribution of data mining methods to this important and challenging field.

## Materials and Methods

### Catalog Description

**Fig 1:**
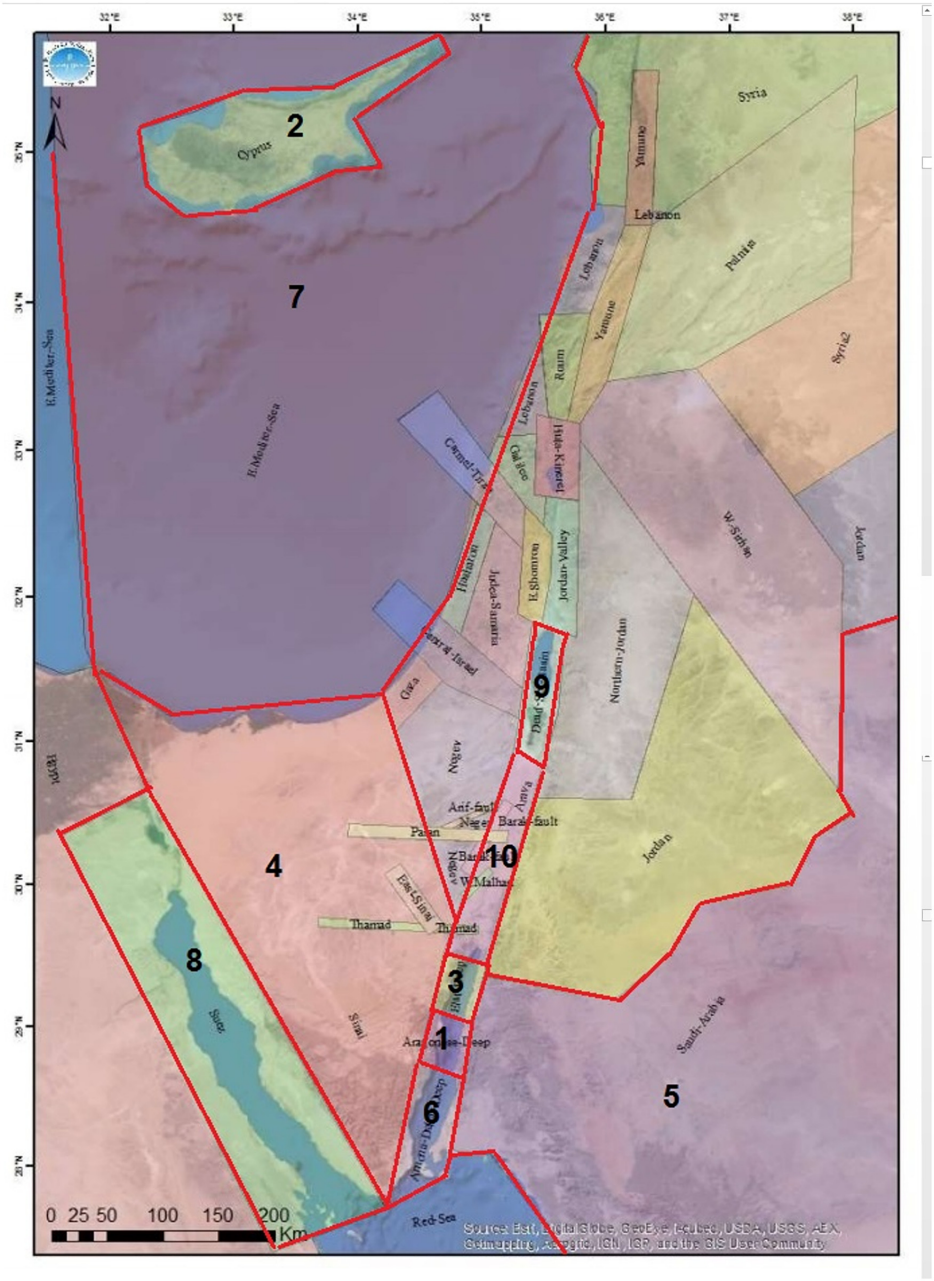
Seismogenic Zones [12]. The top 10 areas are marked with numbers.

We have obtained 9,531 instrumental records of earthquake events, which took place between 05/01/1900 and 07/08/2011. These records are stored in the Geophysical Institute of Israel (GII) database (www.gii.co.il) and they are available from the Institute upon request. Each record includes the following information:

1. Earthquake timing (YEAR, MONTH, DAY, HOUR, MINUTE, and SECOND)
2. Earthquake magnitude expressed by the following three parameters:
  - *Md*—Local Richter Magnitude
  - *Mb*–Estimated Body Waves Magnitude
  - *Mm*–Seismic Moment Magnitude
3. Earthquake location (X, Y, H, LAT(N), and LON (E))
4. AREA (33 distinct areas defined by the Geophysical Institute of Israel). Details on the boundaries and the characteristics of seismogenic zones in Israel are available in [12]. The map of seismogenic zones is shown in Fig 1.
5. FELT (F—felt, blank—not felt)
6. MSK (The maximum measured magnitude in Israel on the MSK, Medvedev-Sponheuer-Karnik, scale)

The dataset includes only events where at least one of the magnitudes (*Md*, *Mb* or *Mm*) has reached the level of 2.0 and higher. Thus, we have computed the maximal magnitude of each event as the maximum of its three magnitudes (*Md*, *Mb*, and *Mm*). The calculated attribute is called *Max_Event_Magnitude*. Based on the website documentation, we have assumed the GII catalog to be complete with respect to this threshold since 1983, leaving us with 9,042 earthquake events to be used in our analysis. In our analysis, we have focused on top 10 areas having the highest number of earthquakes, which cover around 80% of all recorded earthquakes. The list of these areas is presented in Table 1 and their geographic location is shown in Fig 1. The areas are connected to each other and they cover part of the Syrian African Fault, the Sinai Peninsula, the Gulf of Suez, and the East Mediterranean (including Cyprus). The second column of Table 1 shows the total number of recorded seismic events, whereas the third and the fourth columns present the maximum and the medium magnitude, respectively, recorded in each area during the period of study. The total number of mainshocks in each area is shown in the last column.

**Table 1:** Top 10 Areas.

No	AREA	Total Amount of Events	Max. Magnitude	Median Magnitude	Number of Mainshocks
**1**	**Aragonese**	1.1.1.1.1.1.1.1 1775	1.1.1.1.1.1.1.2 6.2	1.1.1.1.1.1.1.3 4.2	1.1.1.1.1.1.1.4 208
**2**	**Cyprus**	1.1.1.1.1.1.1.5 1412	1.1.1.1.1.1.1.6 6.1	1.1.1.1.1.1.1.7 4.3	1.1.1.1.1.1.1.8 376
**3**	**Elat Deep**	1.1.1.1.1.1.1.9 1365	1.1.1.1.1.1.1.10 5.3	3.35	1.1.1.1.1.1.1.11 154
**4**	**Sinai**	1.1.1.1.1.1.1.12 526	1.1.1.1.1.1.1.13 5	1.1.1.1.1.1.1.14 3.5	1.1.1.1.1.1.1.15 145
**5**	**Saudi Arabia**	1.1.1.1.1.1.1.16 457	1.1.1.1.1.1.1.17 5.8	1.1.1.1.1.1.1.18 4.1	1.1.1.1.1.1.1.19 139
**6**	**Arnona Dakar**	1.1.1.1.1.1.1.20 454	1.1.1.1.1.1.1.21 5.5	1.1.1.1.1.1.1.22 3.4	1.1.1.1.1.1.1.23 110
**7**	**East Mediterranean Sea**	1.1.1.1.1.1.1.24 446	1.1.1.1.1.1.1.25 5.5	1.1.1.1.1.1.1.26 4.2	1.1.1.1.1.1.1.27 282
**8**	**Suez**	1.1.1.1.1.1.1.28 359	1.1.1.1.1.1.1.29 5.4	1.1.1.1.1.1.1.30 4.3	225
**9**	**Dead Sea**	1.1.1.1.1.1.1.31 341	1.1.1.1.1.1.1.32 5.2	1.1.1.1.1.1.1.33 3.5	1.1.1.1.1.1.1.34 203
**10**	**Arava Valley**	1.1.1.1.1.1.1.35 306	1.1.1.1.1.1.1.36 4.3	1.1.1.1.1.1.1.37 3.4	1.1.1.1.1.1.1.38 191

### Data Cleaning

It is a common practice to remove foreshock and aftershock events before computing various seismic indicators [9]. An *aftershock* is defined as a minor shock following the mainshock of an earthquake whereas a *foreshock* is an earthquake followed by an event of equal or larger magnitude within a short period of time [13]. Since the time windows for both foreshock and aftershock removal are usually measured in days [13], we have replaced multiple events recorded on the same day by a single “daily event” having the maximum magnitude on that day. No significant information is lost by this operation, since all events of lower magnitude, which occurred on the same day, can be safely considered as either foreshocks or aftershocks of the maximum magnitude event. Based on [13], we have defined Algorithm 1 for foreshock and aftershock removal, which is applied to a set of daily seismic events in a given area. The algorithm assumes the set of events to be sorted in the order of their occurrence.

**Algorithm 1.** Foreshock and aftershock removal

Input:

 *Fore_Shock_Win* **//the size of the foreshock window (days**)

 *After_Shock_Win* **//the size of the aftershock window (days)**

 *Num_Records* **//number of daily events in a given area**

 *Max_of_Max [k]* **//magnitude of daily event *k***

 *Date [k]* **//date of daily event *k***

Output: *Shock[k]* **// shock type of daily event *k* (*S*–mainshock, *F*–foreshock, *A*–aftershock)**

For (*k* = 0; *k* < *Num_Records*; *k*++)

 *Shock* [*k*] ← *S* **//default = mainshock**

 **//Identify foreshocks**

 If (*k* < (*Num_Records*—1))

  *Next* ← *k* + 1

  *Diff_Next* ← (*Date[Next]*—*Date[k]*) **//Compute difference to the next record suspicious as mainshock**

  *Diff[k]* ← *Diff_Next*

  While ((*Diff_Next* ≤ *Fore_Shock_Win*) and (*Shock*[*k*] = *S*) and (*Next* < *Num_Records*))

   If (*Max_of_Max*[*Next*] ≥ *Max_of_Max*[*k*])

    *Shock*[*k*] ← *F*//foreshock

    *Foreshocks* ← *Foreshocks* + 1

   Else

    *Next*++

    If (*Next* < *Num_Records*)

     *Diff_Next* ← (*Date*[*Next*]—*Date*[*k*]) **//Compute difference to the next suspicious record**

    End If

   End If

  End While

 End If

 **//Identify aftershocks**

 If (*k* > 0)

  *Previous* ← *k*—1

  *Diff_Previous* ← (*Date*[*k*]—*Date*[*Previous*]) **//Compute difference to the previous record suspicious as mainshock**

  While ((*Diff_Previous* ≤ *After_Shock_Win*) and (*Shock*[*k*] ≠ *A*) and *Previous* ≥ 0)

   If (*Max_of_Max*[*Previous*] > *Max_of_Max*[*k*])

    *Shock*[*k*] ← *A* **//aftershock**

    *After_Shocks* ← *After_Shocks* + 1

   Else

    *Previous* ← *Previous*—1

    If (*Previous* ≥ 0)

     *Diff_Previous* = (*Date*[k]—*Date*[*Previous*]) **//Compute difference to the previous suspicious record**

    End If

   End If

  End While

 End If

End For **//Next record**

Following the foreshock and aftershock studies presented in [13] and [14], we have set the sizes of the foreshock and the aftershock windows to 5 and 10 days, respectively. The number of mainshocks identified in each area by Algorithm 1 is shown in the fifth column of Table 1.

### Seismicity Indicators Extraction

The ultimate goal of this study is to predict the maximum earthquake magnitude in a given area during the next year. Thus the feature vector can include any seismic indicator, which can be calculated at the end of the current year. We start with the description of the six indicators adopted from [4] and then present some additional indicators, which were not used in the previous studies.

#### Existing Indicators [4]

All six indicators of [4] are calculated over a sliding window of the last *n* events in a given area preceding the forecasted period. The minimal value of *n* used in various studies is 50 (e.g., [4] [9]) and this is also the value we have used here. The value of *n* determines the first period in a given catalog (a year in our case), for which the seismic indicators can be estimated and the forecast can be made. Due to the limited amount of complete data in the GII catalog (about 28 years only), we have not experimented with values of *n*, which are larger than 50. As indicated in Table 1, we have limited our analysis to top 10 areas that experienced at least 110 mainshocks. The definitions of the specific indicators follow.

1. The *T* value. This is the time elapsed over the last *n* events of magnitude greater than a predefined threshold value. It is defined as
  $$T=t_{n}-t_{1}$$ (2)
  where *t*_*n*_ is the time of occurrence of the *n*-th event and *t*_*1*_ is the time of occurrence of the first event. If there is an increase in the seismic activity during the period preceding the forecasted year, the *T* value becomes smaller and vice versa. The threshold value used in our study is the completeness threshold of the GII catalog (Richter magnitude of 2.0).
2. The Mean Magnitude. This is the mean of the Richter magnitudes of the last *n* events defined as
  $$M_{\text{mean}}=\frac{\sum_{i=1}^{n}M_{i}}{n}$$ (3)
  This is another important indicator of recent seismic activity.
3. The rate of square root of seismic energy released (*dE*^1/2^). The rate of square root of seismic energy released over time *T* is defined as
  $$dE^{1/2}=\frac{\sum E^{1/2}}{T}$$ (4)
  where *E*^*1/2*^ is the square root of the seismic energy (*E*) is calculated from the corresponding Richter magnitude *M* using the following empirical relationship [15]:
  $$E=10^{11.8+1.5M}\text{erg s}$$ (5)
  If the release of seismic energy is disrupted for significantly long periods of time (called "seismic quiescence"), the accumulated energy will be released abruptly in the form of a major seismic event when the stored energy reaches a threshold [16].
4. The slope of the regression line fitting the curve of the log of the earthquake frequency versus magnitude (*b* value). This parameter is based on the Gutenberg-Richter inverse power law (Eq 1) and it can be calculated using Algorithm 2 from the last *n* events sorted in the order of their occurrence. The algorithm calls a standard **Linear_Regression** function, which implements the least squares linear regression method with the following input parameters:
  *n*–the sample size (number of observations)
  *Intercept*– 0 if the equation intercept (*a*) should be equal to zero and 1 otherwise
  *y*–*n* values of the dependent variable (log_10_ *N*(*M)* in our case)
  *x*—*n* values of the independent variable (*M* in our case)
  More details on calculating the coefficients of the linear regression equation with the least squares method are available in [17].
  **Algorithm 2.** Calculating the Gutenberg-Richter relationship
  Input:
   *Min_Shock_No* **//ID of the first mainshock in the sliding window of last events**
   *Max_Shock_No* **//ID of the last mainshock in the sliding window of last events**
   *Shock_Max_of_Max[m]* **//Magnitude of mainshock *m***
  Output:
   *a*, *b* **//Coefficients of** Eq (1)
   *Tot_Shocks* **//Size of the regression sample**
  Tot_Shocks = Max_Shock_No—Min_Shock_No + 1
  *j* ← 0 **//Initialize the index of the regression sample**
  For (*l* = *Min_Shock_No*; *l* ≤ *Max_Shock_No*; *l*++)
    *N_Sample [j]* ← 0 **//initialize *N (M)*–the number of events with magnitude greater or equal to the magnitude of event *j***
    *Shock_Max_of_Max_Sample* [*j*] ← *Shock_Max_of_Max*[*l*]; **// copy the mainshocks subset to the regression sample**
    For (*m* = *Min_Shock_No*; *m* ≤ *Max_Shock_No*; *m*++)
     If (*Shock_Max_of_Max*[*m*] ≥ *Shock_Max_of_Max*[*l*])
      *N_sample* [*j*] ← *N_sample* [*j*] + 1 **//increment number of events with magnitude greater or equal to the event *l***
     End If
    End For
    *j ← j + 1* **// Increment the index of the regression sample**
  End For
  **Linear_Regression.** (*Tot_Shocks*, 1, log_10_ (*N_Sample*), *Shock_Max_of_Max_Sample*) **//Find the coefficients *a* and *b* of the linear regression equation using the least squares method with non-zero intercept**
5. The Mean Squared Error (MSE) of the regression line based on the Gutenberg-Richter inverse power law (*η* value). We use here an unbiased estimator of this parameter, which is defined as follows:
  $$\eta =\frac{\sum {\left({\text{log}}_{10}N_{i}-(a-bM_{i})\right)}^{2}}{n-1}$$ (6)
  Where *N*_*i*_ is the number of events in the sliding window with magnitude *M*_*i*_ or greater. This is a measure of the conformance of the observed seismic data to the Gutenberg-Richter inverse power-law relationship.
6. Magnitude deficit or the difference between the largest observed magnitude and the largest expected magnitude based on the Gutenberg-Richter relationship (*ΔM* value). It is shown in [4]) that this value is equal to the ratio *a/b* of the Eq (1) coefficients.

The work of [4] has used two additional seismic indicators: Mean time between characteristic events (large earthquakes) and Coefficient of variation of the mean time between characteristic events. We have excluded these indicators from our study for two reasons: the short period of the available complete data, which includes a small number of large earthquakes if any, and the limited performance of these two features reported in [4].

#### New Indicators

In addition to the seismic indicators (1)–(6) adopted from the existing earthquake prediction literature, we have defined two new feature types based on the Gutenberg-Richter law *during the forecasted period* (a year in our case). Our goal is to predict whether the maximum earthquake magnitude in the following year will exceed some magnitude threshold (e.g., the median of maximum yearly magnitudes in the same area). Eq (1) implies that the expected number of events exceeding a threshold *th* during the forecasted period can be calculated by:

$$N(M\geq th)=10^{a-b*th}$$ (7)

Consequently, the probability that the magnitude of a randomly selected event will exceed the threshold *th* can be found by the ratio between *N(M ≥ th)* and the expected number of recorded events *N(M ≥ M*_*0*_*)*, where *M*_*0*_ is the completeness threshold of the catalog (2.0 in case of the GII database). This probability is derived below:

$$\text{Prob}(M\geq th)=\frac{10^{a-b*th}}{10^{a-b*M_{0}}}=10^{-b(th-M_{0})}$$ (8)

From Eq (8), we can find the probability that the maximum magnitude of *n* events recorded in the forecasted period will exceed the threshold as a complement of the probability that the magnitude of all *n* events will be below the threshold:

$$\text{Prob }(\text{Max}\geq th)=1-{\left(\text{Prob }(M<th)\right)}^{n}=1-{\left(1-10^{-b(th-M_{0})}\right)}^{n}$$ (9)

Unfortunately, Eq (9) cannot be used directly for estimating the probability of the maximum earthquake magnitude to exceed the threshold *th*, since the number of events in the forecasted period is not known in advance and we are not aware of any reliable models predicting this number. Instead, we try to utilize the recurrence patterns potentially presenting in the yearly earthquake data by calculating the moving averages of the yearly number of events over 1–10 years preceding the forecasted year and apply Eq (9) to each of these moving averages. Thus we obtain 20 new seismic indicators: *MA* (1)–*MA* (10) and *Prob_Max* (1)–*Prob_Max* (10), where *MA (x)* stands for the moving average of the number of events (mainshocks) over *x* years and *Prob_Max (x)* is the probability that the maximum magnitude of *MA (x)* events will exceed the threshold *th*. Consequently, we have increased the number of predictive features from six to twenty-six.

### Earthquake Prediction Algorithms and Tools

We define the earthquake magnitude prediction as a binary classification task based on the median of maximum yearly magnitudes in a given area. According to our approach, the forecasted year is labeled as belonging to one of two classes: "Yes" if the maximum earthquake magnitude exceeds the median or "No" if it is below the median. We have chosen the median magnitude as the prediction threshold, since it presents the most balanced classification task and also due to the fact that the median magnitudes in most areas under study fall in the range of [3.5, 4.2] making the difference between earthquakes which rarely cause any damage and the earthquakes where some extent of damage should be expected. We assume that all seismic indicators defined in the previous section are calculated at the end of the previous year, which also serves as the end-point of the sliding window of *n* last events.

Due to the large number of available seismic indicators [26], we have evaluated the predictive effect of each indicator using a popular feature selection metric called Information Gain Ratio (IGR) [18], which “punishes” the multi-valued attributes via dividing their information gain by their own entropy (“Split Information”). The Information Gain Ratios of seismic indicators were calculated and normalized in the [0, 1] range using the Weight by Information Gain Ratio operator of RapidMiner [19]. As indicated below, when using classification methods, which do not have a built-in feature selection property (k-NN, ANN, and SVM), we have removed the attributes with normalized weights below 0.8 using the Select by Weights RapidMiner operator.

The following classification algorithms were used in our experiments:

- J48, the Java implementation of the most popular decision-tree algorithm C4.5 [18]. As C4.5 chooses the best testing feature at each tree node, the algorithm was used without feature selection.
- AdaBoost [20], a well-known meta-learner algorithm, which repeatedly applies a weak classifier to weighted examples in a dataset. At each step, the weights of the examples are updated to put more focus on the incorrectly classified examples. Following relatively poor results with J48 (see below), we have used AdaBoost in conjunction with J48. No feature selection was applied.
- Information Network (IN) [21], a decision-tree algorithm, which uses the same feature across the nodes of a given layer. The features are selected incrementally to maximize a global decrease in the conditional entropy of the classification attribute. The IN induction algorithm is using the pre-pruning approach: when no attribute causes a statistically significant decrease in the entropy, the model construction is stopped. In [21], the algorithm is shown empirically to produce much more compact models than other methods of decision-tree learning, while preserving nearly the same level of classification accuracy.
- Multi-Objective Info-Fuzzy Network (M-IFN) [22], a multi-objective extension of the IN model, where each leaf node is associated with several target (predicted) attributes. The M-IFN algorithm was shown theoretically and empirically to produce the optimal (most accurate) models when all target attributes are either mutually independent or completely dependent on each other. This property makes it particularly useful for trying to simultaneously predict two target attributes, the maximum magnitude and the total number of seismic events, which are known to be related to each other by the Gutenberg-Richter law. Both IN and M-IFN have the built-in feature selection property. Both algorithms have only one tuning parameter, the significance level, which has a default value of 0.001.
- K-Nearest Neighbors (k-NN) [23], a “lazy” learning algorithm, which classifies each testing example by the labels of *k* training examples that are most similar to it. To avoid the impact of irrelevant features on distance calculations, we have applied this algorithm with feature selection by removing the attributes with normalized weights below 0.8. The best value of *k* was chosen to optimize the classification performance (*k* = 2 for all features and *k* = 3 for the basic features).
- Support Vector Machine (SVM) [24], a powerful classification algorithm, which searches for the optimal separating hyperplane between two classes. We have chosen SVM with RBF kernel, since it optimized the classification performance. Feature selection was applied with the same threshold (0.8).
- Artificial Neural Networks (ANN) [23], a biologically inspired method for learning complex, nonlinear functions. We have experimented with two ANN learning algorithms: Backpropagation (BP) and Gaussian radial basis function network (RBF). The attributes with normalized weights below 0.8 were removed.

All above classification algorithms, except IN and M-IFN, were applied using the appropriate RapidMiner operators. For IN and M-IFN, we have used our own implementation of the algorithms, which can be downloaded from doi:10.5061/dryad.9tq97. Unless indicated above, the default settings of all algorithms, set by RapidMiner or by the IN / M-IFN software, were kept unchanged.

## Results

Rather than training separate classification models on small datasets related to each seismic area, we have combined the data of 10 areas into one data table to produce a general classification model. Since the analyzed catalog includes only 28 years of complete data, we have kept the last five annual records of each area (referring to the years 2006–2010) for testing while using the previous annual records for training. The class label of each record was set to zero if the maximum magnitude in the corresponding year and area was below the area median, and set to one otherwise. When running the M-IFN algorithm, we have added a second target attribute to each yearly record—the number of mainshocks. Our training set included 136 records, whereas the testing set had 49 records. The years, where no earthquakes were recorded in a given area (e.g., Sinai in 2010), were excluded from the analysis. The preprocessed training and testing datasets in the IN / M-IFN compatible format can be downloaded from doi:10.5061/dryad.9tq97.

The Information Gain Ratio (IGR) weights of all features are shown in Table 2, in the ascending order of importance. It is noteworthy that the six moving average features have taken the highest rankings [21–26], followed by six magnitude exceeding probabilities (ranks 15–20). The *b* coefficient of the Gutenberg-Richter equation is ranked only 14 out of 26, whereas other seismic indicators of [4] are ranked between 5 and 12 only.

**Table 2:** Information Gain Ratio weights of all features (*—selected features).

Feature	Type	IGR Weight	Normalized IGR Weight	Rank
**MA (2)**	1.1.1.1.1.1.1.39 New	1.1.1.1.1.1.1.40 0.082	1.1.1.1.1.1.1.41 0.336	1.1.1.1.1.1.1.42 1
**MA (3)**	1.1.1.1.1.1.1.43 New	1.1.1.1.1.1.1.44 0.082	1.1.1.1.1.1.1.45 0.336	1.1.1.1.1.1.1.46 2
**MA (5)**	1.1.1.1.1.1.1.47 New	1.1.1.1.1.1.1.48 0.082	1.1.1.1.1.1.1.49 0.336	1.1.1.1.1.1.1.50 3
**MA (9)**	1.1.1.1.1.1.1.51 New	1.1.1.1.1.1.1.52 0.082	1.1.1.1.1.1.1.53 0.336	1.1.1.1.1.1.1.54 4
**M_mean**	1.1.1.1.1.1.1.55 Basic	1.1.1.1.1.1.1.56 0.082	1.1.1.1.1.1.1.57 0.336	1.1.1.1.1.1.1.58 5
**Mean Square**	1.1.1.1.1.1.1.59 Basic	1.1.1.1.1.1.1.60 0.082	1.1.1.1.1.1.1.61 0.336	1.1.1.1.1.1.1.62 6
**Rate of Energy**	1.1.1.1.1.1.1.63 Basic	1.1.1.1.1.1.1.64 0.090	1.1.1.1.1.1.1.65 0.375	1.1.1.1.1.1.1.66 7
**Prob_Max (8)**	1.1.1.1.1.1.1.67 New	1.1.1.1.1.1.1.68 0.096	1.1.1.1.1.1.1.69 0.409	1.1.1.1.1.1.1.70 8
**Prob_Max (9)**	1.1.1.1.1.1.1.71 New	1.1.1.1.1.1.1.72 0.096	1.1.1.1.1.1.1.73 0.409	1.1.1.1.1.1.1.74 9
**Delta M**	1.1.1.1.1.1.1.75 Basic	1.1.1.1.1.1.1.76 0.096	1.1.1.1.1.1.1.77 0.409	1.1.1.1.1.1.1.78 10
**Prob_Max (7)**	1.1.1.1.1.1.1.79 New	1.1.1.1.1.1.1.80 0.101	1.1.1.1.1.1.1.81 0.438	1.1.1.1.1.1.1.82 11
**T Elapsed**	1.1.1.1.1.1.1.83 Basic	1.1.1.1.1.1.1.84 0.101	1.1.1.1.1.1.1.85 0.438	1.1.1.1.1.1.1.86 12
**Prob_Max (10)**	1.1.1.1.1.1.1.87 New	0.106	1.1.1.1.1.1.1.88 0.466	1.1.1.1.1.1.1.89 13
**b**	1.1.1.1.1.1.1.90 Basic	1.1.1.1.1.1.1.91 0.111	1.1.1.1.1.1.1.92 0.491	1.1.1.1.1.1.1.93 14
**Prob_Max (5)**	1.1.1.1.1.1.1.94 New	1.1.1.1.1.1.1.95 0.111	1.1.1.1.1.1.1.96 0.491	1.1.1.1.1.1.1.97 15
**Prob_Max (6)**	1.1.1.1.1.1.1.98 New	1.1.1.1.1.1.1.99 0.111	1.1.1.1.1.1.1.100 0.491	1.1.1.1.1.1.1.101 16
**Prob_Max (1)**	1.1.1.1.1.1.1.102 New	1.1.1.1.1.1.1.103 0.124	1.1.1.1.1.1.1.104 0.562	1.1.1.1.1.1.1.105 17
**Prob_Max (4)**	1.1.1.1.1.1.1.106 New	1.1.1.1.1.1.1.107 0.129	1.1.1.1.1.1.1.108 0.584	1.1.1.1.1.1.1.109 18
**Prob_Max (3)**	1.1.1.1.1.1.1.110 New	1.1.1.1.1.1.1.111 0.133	1.1.1.1.1.1.1.112 0.606	1.1.1.1.1.1.1.113 19
**Prob_Max (2)**	1.1.1.1.1.1.1.114 New	1.1.1.1.1.1.1.115 0.137	1.1.1.1.1.1.1.116 0.628	1.1.1.1.1.1.1.117 20
MA (1) *	1.1.1.1.1.1.1.118 New	1.1.1.1.1.1.1.119 0.181	1.1.1.1.1.1.1.120 0.864	1.1.1.1.1.1.1.121 21
MA (4) *	1.1.1.1.1.1.1.122 New	1.1.1.1.1.1.1.123 0.181	1.1.1.1.1.1.1.124 0.864	1.1.1.1.1.1.1.125 22
MA (6) *	1.1.1.1.1.1.1.126 New	1.1.1.1.1.1.1.127 0.181	1.1.1.1.1.1.1.128 0.864	1.1.1.1.1.1.1.129 23
MA (7) *	1.1.1.1.1.1.1.130 New	1.1.1.1.1.1.1.131 0.181	1.1.1.1.1.1.1.132 0.864	24
MA (8) *	1.1.1.1.1.1.1.133 New	1.1.1.1.1.1.1.134 0.181	1.1.1.1.1.1.1.135 0.864	1.1.1.1.1.1.1.136 25
MA (10) *	1.1.1.1.1.1.1.137 New	0.207	1.1.1.1.1.1.1.138 1.000	1.1.1.1.1.1.1.139 26

Since the maximum magnitude prediction is a probability estimation problem, we have used the area under testing ROC curves (Testing AUC) for evaluating different classification algorithms. The ROC (Receiver Operating Characteristics) curves [25] are two-dimensional graphs in which the TP (True Positive) rate is plotted on the Y axis and the FP (False Positive) rate is plotted on the X axis. In the case of earthquake prediction models, an ROC curve depicts the relative trade-off between true positives (years which exceeded the median magnitude and were predicted in advance) and false positives (unnecessary warnings issued for the years which did not exceed the median). In any ROC curve, the diagonal line y = x represents the strategy of randomly guessing a class. A useful classifier should have an ROC curve above the diagonal line implying that its AUC is higher than 0.5. An ideal classifier, which is never wrong in its prediction, would have the maximum AUC of 1.0.

To further evaluate the contribution of the 20 new seismic indicators (moving averages over 1–10 years and the corresponding median exceeding probabilities), we have performed two sets of experiments with all classification algorithms: using all 26 features and using only the six basic features from [4]. The testing AUC results are presented in Table 3. The multi-objective model of M-IFN clearly shows the best result (AUC = 0.698), explained by its capability to take into account the relationship between two target variables: the number of earthquakes and the maximum earthquake magnitude during the same year. Such multi-target classification capability is missing in all other classification algorithms included in our experiments. SVM and RBF-based neural network are ranked second and third, respectively. It is evident that the new features provide a significant improvement in the classification performance over the basic features of [4] for all tested classification algorithms. Some induced models were as good as a random guess (e.g., J48 with basic features) or even worse (like BP-NN with all features).

**Table 3:** Testing AUC Results. The best values are shown in bold.

Algorithm	All features	Basic Features
**J48**	0.549	0.500
**J48-Adaboost**	0.631	0.500
**IFN**	0.585	0.500
**M-IFN**	**0.698**	0.394
**k-NN**	0.628	**0.532**
**SVM (nu-SVC)**	0.675	0.529
**BP-NN**	0.461	0.456
**W-RBF**	0.655	0.485

The M-IFN algorithm, which has induced the most accurate prediction model, has selected the following predictive features:

- MA (1)–the number of events (mainshocks) in the last year
- Prob_Max (3)—the probability that the maximum magnitude of MA (3) events will exceed the median
- Prob_Max (7)—the probability that the maximum magnitude of MA (7) events will exceed the median

The M-IFN prediction rules are shown in Table 4. The rules with the highest probability of the maximum earthquake magnitude to exceed the median (Rules 2, 5, and 7) refer either directly, or indirectly to an increase in the number of events during the preceding one, three and seven years, respectively. This result agrees with the *precursory scale increase phenomenon*, which involves an increase in the magnitude and rate of occurrence of minor earthquakes as a precursor of a major seismic event [26].

**Table 4:** M-IFN Prediction Rules.

Rule No.	Condition	Prob. (Max M > Median)
0	If MA (1) is between 6 and 10	0.674
1	If MA (1) is between 15 and 21	0.643
2	If MA (1) is more than 21	0.800
3	If MA (1) is between 0 and 6 and Prob. to exceed median (3) is between 0.201 and 0.431	0.583
4	If MA (1) is between 0 and 6 and Prob. to exceed median (3) is between 0.431 and 0.711	0.385
5	If MA (1) is between 0 and 6 and Prob. to exceed median (3) is more than 0.711	0.857
6	If MA (1) is between 10 and 15 and Prob. to exceed median (3) is between 0.201 and 0.431	0.667
7	If MA (1) is between 10 and 15 and Prob. to exceed median (3) is more than 0.711	1.000
8	If MA (1) is between 10 and 15 and Prob. to exceed median (3) is between 0.431 and 0.711 and Prob. to exceed median (7) is between 0.326 and 0.643	0.714
9	If MA (1) is between 10 and 15 and Prob. to exceed median (3) is between 0.431 and 0.711 and Prob. to exceed median (7) is more than 0.643	0.000

**Fig 2:**
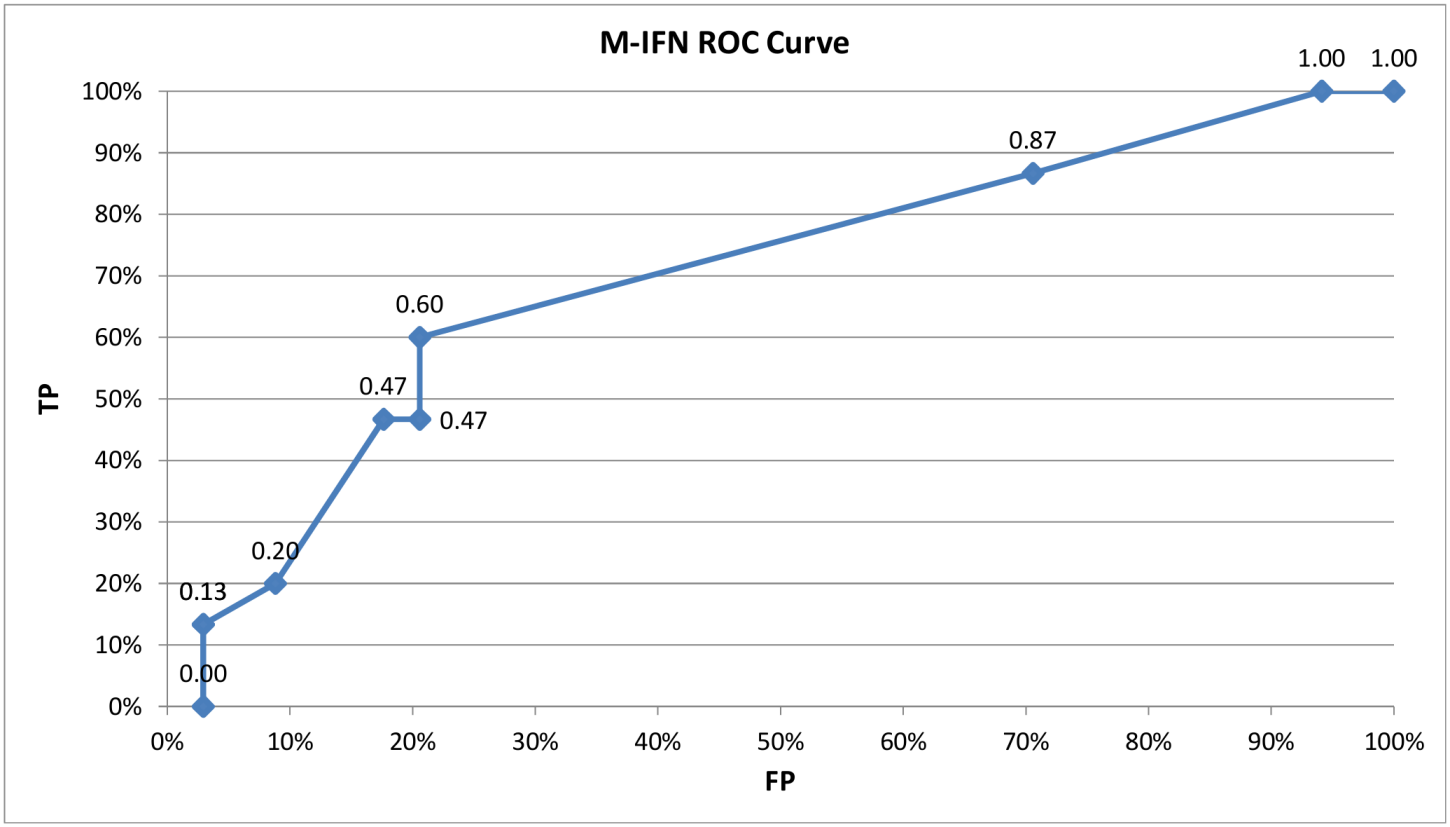
The M-IFN ROC Curve

The M-IFN Testing ROC curve is shown in Fig 2. The Youden Index [27] value of the curve (max (*tp—fp*)) is 39.41% corresponding to sensitivity of 60.00%, specificity of 79.41%, and false alarm rate of 20.59%.

## Conclusion

The results of this work demonstrate the potential of data mining methods for the important task of earthquake prediction in seismically active areas around the world. As opposed to previously published long-term forecasting methods, such as [7] and [8], which build mainly upon the Gutenberg-Richter inverse power law, we show in this paper that the data mining models can utilize multiple seismic indicators as predictive features. The best performing model in this study, Multi-Objective Info-Fuzzy Network (M-IFN), does not require any fine-tuning of its parameters, unlike the clustering approach presented in [9], which involves selecting the optimal number of clusters. The specificity of the M-IFN model over the 10 analyzed areas (79.41%) is close to the specificity of one of the two area models induced by [9] from the Spanish seismic dataset (82.56%), whereas the M-IFN sensitivity (60.00%) is lower by 20%–30% than the sensitivity of the two area models in [9]. Compared to the short-term forecasting study in [4], which has reported the sensitivity / recall of 0.44–0.67 and the false alarm rate of 0.31–0.44, the M-IFN sensitivity of 0.60 corresponds to the false alarm rate of 0.21 only.

Possible ways for further improving the accuracy of data mining models include taking into account spatio-temporal associations between neighboring seismic areas (similar to [8]) and applying time-series forecasting models for predicting the number and the magnitude of seismic events in subsequent periods.
