# Correction: Predicting the Maximum Earthquake Magnitude from Seismic Data in Israel and Its Neighboring Countries

**DOI:** 10.1371/journal.pone.0151751

**Published:** 2016-03-14

**Authors:** The PLOS ONE Staff

Tables 1 and 2 appear incorrectly in the published article. Please see the correct Tables 1 and 2, and their captions here. The publisher apologizes for the errors.

**Table 1:** Top 10 Areas.

No	AREA	Total Amount of Events	Max. Magnitude	Median Magnitude	Number of Mainshocks
**1**	**Aragonese**	1775	6.2	4.2	208
**2**	**Cyprus**	1412	6.1	4.3	376
**3**	**Elat Deep**	1365	5.3	3.35	154
**4**	**Sinai**	526	5	3.5	145
**5**	**Saudi Arabia**	457	5.8	4.1	139
**6**	**Arnona Dakar**	454	5.5	3.4	110
**7**	**East Mediterranean Sea**	446	5.5	4.2	282
**8**	**Suez**	359	5.4	4.3	225
**9**	**Dead Sea**	341	5.2	3.5	203
**10**	**Arava Valley**	306	4.3	3.4	191

**Table 2:** Information Gain Ratio weights of all features (*—selected features).

Feature	Type	IGR Weight	Normalized IGR Weight	Rank
**MA (2)**	New	0.082	0.336	1
**MA (3)**	New	0.082	0.336	2
**MA (5)**	New	0.082	0.336	3
**MA (9)**	New	0.082	0.336	4
**M_mean**	Basic	0.082	0.336	5
**Mean Square**	Basic	0.082	0.336	6
**Rate of Energy**	Basic	0.090	0.375	7
**Prob_Max (8)**	New	0.096	0.409	8
**Prob_Max (9)**	New	0.096	0.409	9
**Delta M**	Basic	0.096	0.409	10
**Prob_Max (7)**	New	0.101	0.438	11
**T Elapsed**	Basic	0.101	0.438	12
**Prob_Max (10)**	New	0.106	0.466	13
**b**	Basic	0.111	0.491	14
**Prob_Max (5)**	New	0.111	0.491	15
**Prob_Max (6)**	New	0.111	0.491	16
**Prob_Max (1)**	New	0.124	0.562	17
**Prob_Max (4)**	New	0.129	0.584	18
**Prob_Max (3)**	New	0.133	0.606	19
**Prob_Max (2)**	New	0.137	0.628	20
MA (1) *	New	0.181	0.864	21
MA (4) *	New	0.181	0.864	22
MA (6) *	New	0.181	0.864	23
MA (7) *	New	0.181	0.864	24
MA (8) *	New	0.181	0.864	25
MA (10) *	New	0.207	1.000	26
